# Routine Use of a Pocket-Sized Handheld Echoscopic Device Plus a Biomarker by Emergency Medicine Residents with an Early Screening Algorithm for Suspected Type A Acute Aortic Syndrome

**DOI:** 10.3390/jcm12041346

**Published:** 2023-02-08

**Authors:** Rui Lian, Tongzhe Zhang, Juanjuan Liu, Guochao Zhang, Tianpeng Hu, Guonan Li, Suqiao Zhang, Guoqiang Zhang

**Affiliations:** 1 Emergency Department, China-Japan Friendship Hospital, Beijing 100029, China; 2 Emergency Department, Lanzhou University Second Hospital, Lanzhou 730030, China; 3 General Surgery Department, China-Japan Friendship Hospital, Beijing 100029, China; 4 Graduate School, Peking Uninon Medical College, Beijing 100730, China

**Keywords:** acute aortic syndrome, handheld echocardiographic devices, emergency medicine resident, ascending aorta diameter, acidic calponin

## Abstract

(1) Background: The early screening strategy for type A acute aortic syndrome (A-AAS) patients has always been challenging. (2) Methods: From September 2020–31 March 2022, 179 consecutive patients with suspected A-AAS were retrospectively reviewed. We assessed the diagnostic value of the use of handheld echocardiographic devices (PHHEs) by emergency medicine (EM) residents either alone or in combination with serum acidic calponin in this patient group. (3) Results: The direct sign of PHHE had a specificity (SP) of 97.7%. The sign of ascending aortic dilatation showed SE = 77.6%, SP = 68.5%, PPV = 48.1% and NPV = 89%. SE, SP, PPV and NPV of a positive PHHE direct sign were 55.6%, 100%, 100% and 71.4% in 19 hypotension/shock patients with suspected A-AAS, respectively. The area under curve (AUC) of acidic calponin combined with an ascending aorta diameter >40 mm was 0.927, with an SE and SP of 83.7% and 89.2%, respectively. These two combined indicators significantly improved the diagnostic efficiency of A-AAS compared with either of them alone (*p =* 0.017; standard error 0.016, Z value 2.39; *p =* 0.001, standard error 0.028, Z value 3.29). (4) Conclusion: EM resident-performed PHHE was highly indicative of A-AAS in patients presenting with shock or hypotension. An ascending aorta diameter > 40 mm combined with acidic calponin demonstrated acceptable diagnostic accuracy as a rapid first-line triage tool to identify patients with suspected A-AAS.

## 1. Introduction

Acute aortic syndrome (AAS) is the most frequently and highly lethal cardiovascular condition in emergency clinics, affecting 4–6 cases/100,000 individuals/year [1]. AAS is attributed to a heterogeneous group of patients with similar clinical characteristics including three underlying pathologic conditions: classic aortic dissection (AD), intramural hematoma (IMH) and penetrating atherosclerotic ulcer (PAU). The Stanford classification system divides AAS into two categories based on whether the ascending aorta (regardless of the site of origin) is involved. In particular, the overall in-hospital mortality rate of type A acute aortic syndrome (A-AAS) is up to 24.4%, owing to a variety of concomitant complications, which is significantly more than that of type B acute aortic syndrome (B-AAS) (10.7%) [2,3]. Moreover, its mortality rate increases by 1–2% per hour after symptom onset without surgical treatment [4]. Therefore, an early diagnosis is essential in order to avoid inappropriate administration of intravenous antithrombotic agent and to provide emergency surgical repair without delay.

Currently, computed tomography angiography (CTA) or transesophageal echocardiography (TOE) is considered the “gold standard” for conclusive diagnosis [1] However, the use of CTA or TOE is costly and frequently requires patient transfer to specialized clinical centers, which introduces great transport risk to hemodynamically unstable patients. Furthermore, CTA, the most frequently used imaging exam for suspected AD, exposes patients to significant radiation and carries inherent risks of anaphylaxis and medium-contrast nephropathy [5,6]. Even with readily available advanced medical conditions, a diagnostic conundrum appears in emergency clinical practice: the rate of erroneous diagnosis remains worryingly high, but the rate of positive CTA performed for suspected AAS is <3% [7,8,9]. In view of the current situation, a bedside clinical triage tool called the aortic dissection detection (ADD) risk score has been proposed as part of the clinical guidelines of the American Heart Association and the American College of Cardiology [10,11]. However, the ADD risk score, which is poorly specific for the diagnosis of AAS, cannot precisely confirm which patients need advanced aortic imaging diagnostic tests [12].

In the setting of ED, standard ultrasonic equipment may be heavy and difficult to handle, whereas pocket-sized handheld echocardiographic devices (PHHEs) carried by emergency physicians (EPs) or emergency medicine (EM) residents for bedside use have emerged in recent years [13,14]. PHHEs can be immediately and safely used for the preliminary evaluation of both direct sonographic signs (intimal flap and intramural hematoma) and indirect sonographic signs of A-AAS (ascending aortic dilatation, pericardial effusion/tamponade and aortic valve insufficiency) at the bedside in the emergency department (ED) [15]. Several plasma biomarkers, such as smooth muscle myosin heavy chain [16], BB isozyme of creatine kinase [17], elastin [18] and D-dimer [19,20,21,22,23] have also been studied as potential candidates as biomarkers for AAS. However, none of these screening tools can be used as a stand-alone test to rule out this deadly disease in all patients. In recent years, a new biomarker called acidic calponin has been studied, demonstrating promising results to diagnose A-AAS in an early stage [24,25]. Calponin, a 34 kDa protein that is a troponin counterpart of smooth muscle has previously been isolated and purified [26]. Calponin has 3 isoforms: acidic, basic and neutral calponin. As a point-of-care test, acidic calponin showed a high specificity in the initial 6 h after symptom onset and may have the potential for use as an early diagnostic biomarker for A-AAS. However, to date, studies about the value of the combined use of a PHHE and acidic calponin for the detection of A-AAS are still lacking. Thus, using CTA as the reference standard, the aim of the study was to evaluate the accuracy of the use of a PHHE by EM residents for the diagnosis of A-AAS. A secondary aim of this study was to identify the diagnostic performance of a combination of a PHHE and acidic calponin.

## 2. Materials and Methods

### 2.1. Study Design

This was a retrospective monocenter diagnostic accuracy study of patients presenting to the ED resuscitation room with suspected A-AAS. The study protocol was approved by the local Ethical Committee of China–Japan friendship Hospital (2016-91). Our department is the Beijing Main Committee Unit for emergency medicine. Since 2000, our medical records have included patients’ consent to use their medical data in clinical research; however, their name does not appear in the informed consent form signed by the patients included in the study.

### 2.2. Study Setting and Enrollment

This study was performed in the ED of a tertiary hospital (1,700,000 emergency attendances per annum). The ED provides CTA and cardiosurgical consultation 24 h/7 days. A total of 3 designated EM residents were contacted in order to perform PHHE as soon as possible after admission. Resident experience in PHHE consisted of a 3-week training course, as well as at least 30 examinations using living models and 6 h of transthoracic ultrasonic image review supervised by an expert before starting the study. Consecutive patients 18 years or older presenting to the ED from 1 September 2019 to 31 March 2021 were retrospectively reviewed in this study if all the following criteria were satisfied: (1) the time from symptom onset to admission was within 24 h; (2) high suspicion of A-AAS based on an ADD risk score > 1, and acute symptoms were not related to any trauma; (3) an alternative diagnosis to A-AAS could not be established by the duty physician after initial medical evaluation; and (4) PHHE could be performed within 30 min of admission in suspected patients without affecting emergent treatment before conclusive diagnosis with CTA.

### 2.3. ADD Risk Score Definition

The ADD risk score covers the following three sections: predisposing conditions (Marfan syndrome/connective tissue disease, family history of aortic disease, history of known aortic valve disease, history of recent aortic manipulation and history of known thoracic aortic aneurysm), pain features (abrupt onset of pain, severe pain intensity and ripping or tearing quality of pain) and physical findings (pulse deficit/systolic blood pressure differential, focal neurological deficit, new murmur of aortic insufficiency and shock state or hypotension).

### 2.4. Machine Used

PHHE transthoracic focus cardiac ultrasound (FOCUS) was carried out using V-Scan (GE Healthcare, Wauwatosa, WI, USA). This PHHE device consists of a display unit (135 × 73 ×28 mm) connected to a broadband-width phased-array probe (1.7–3.8 MHz; 120 × 33 × 26 mm).

### 2.5. EP-Performed FOCUS using PHHE

The patients were examined in a semirecumbent position or left lateral decubitus position. Evaluation views of the aorta included left parasternal, left high parasternal, apical and subcostal views. In patients undergoing FOCUS using PHHE as the first imaging diagnostic test, one direct sign and three indirect signs of A-AAS were observed. The one direct sonographic sign of A-AAS was the presence of an intimal flap separating two aortic lumens or intramural hematoma (circular or crescentic thickening of the aortic wall > 5 mm) in the ascending aorta. Three indirect sonographic signs included ascending aorta diameter > 40 mm, pericardial effusion or tamponade and aortic valve regurgitation detected by color Doppler.

### 2.6. Acidic Calponin Analysis

Venous EDTA-treated blood samples were collected from patients at the time of initial medical evaluation before any surgical procedure in the ED. After centrifuging at 3000 rpm for 15 min at 4 °C, the separated plasma was aliquoted into cryovials, and two tubes were collected for each sample, which were stored at −80 °C until detection. Finally, the levels of serum markers were measured with automated latex agglutination enzyme-linked immunosorbent assay (ELISA) tests according to the standard procedures and protocols. The laboratory technicians were unaware of the clinical data.

### 2.7. Diagnostic Procedures

All the following data were recorded: (1) demographics, medical history, clinical examination and vitals; (2) 12-lead electrocardiogram (ECG); (3) laboratory blood tests (blood routine, blood biochemistry, coagulation function, arterial blood gas analysis, myocardial markers, serum biomarkers and other related tests); and (4) medical imaging (FOCUS using PHHE plus CTA (Canon Aquilion ONE 640)). CTA images were analyzed and reported by consultant radiologists available in the ED 24 h/day. The final diagnosis was established by two independent senior EPs after judging all collected clinical data and aortic imaging studies. Any of the following diagnoses were considered A-AAS: classical Stanford type A AD, IMH of the ascending aorta and PAU of the ascending aorta.

### 2.8. Statistical Analysis

Continuous variables are presented as mean ± SD, and categorical data are presented as numbers and proportions. Intergroup differences were evaluated using an independent two-sample t test, chi-square test or Fisher’s exact test. Diagnostic accuracy was evaluated by sensitivity (SE), specificity (SP), positive and negative predictive value (PPV and NPV, respectively) and accuracy (AC) for dichotomous tests and by the area under the ROC curve (AUC) for quantitative tests. Data were analyzed using GraphPad Prism 7.0 software (GraphPad Inc. San Diego, CA, USA). Corrected values of *p* < 0.05 were considered statistically significant.

## 3. Results

### 3.1. Patient Characteristics

**Figure 1:**
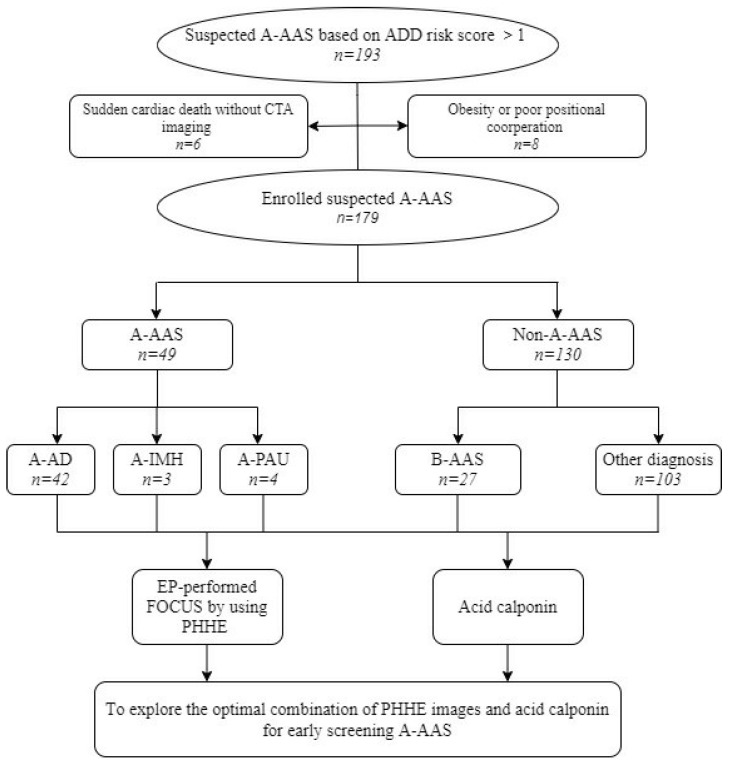
Study flow chart.

During the 18-month study period, a consecutive sample of 193 patients was identified as eligible; in 8 of these patients, physicians were unable to acquire clear PHHE images because of habitus and poor positional cooperation, and 6 did not complete CTA imaging due to sudden cardiac arrest, leaving 179 for analysis. Patient flow and enrollment are shown in Figure 1. A-AAS was ultimately diagnosed in 49 (27.4%) patients: 42 (23.5%) patients had classical A-AD, 3 (1.7%) had an IMH of the ascending aorta and 4 (2.2%) had A-PAU of the ascending aorta. A-AAS was ruled out in 130 (72.6%) patients, with 27 (15.1%) patients presenting with AAS not involving the ascending aorta. The alternative diagnoses were ACS (36 patients, 27.7%), digestive disease (21, 11.7%), non-AAS-related pericardial effusion (12, 6.7%), pulmonary embolism (5, 2.8%), pneumonia (8, 4.5%), Takayasu arteritis (2, 1.1%), non-AAS-related limb pain (8, 4.5%), non-AAS-related AIS (6, 3.4%) and other diagnoses (5, 2.8%). The clinical characteristics and ADD markers in the study patients are summarized in Table 1. Among the 49 patients included in the A-AAS group, 35 were male (71.4%), and 14 were female (28.6%), with a median age of 55.6 (standard deviation (SD) ± 16.7). Of the 130 non-A-AAS cases, 89 were male (68.5%), and 14 were female (10.8%), with a median age of 62.7 (SD ± 13.1). The patients with non-A-AAS were older than the patients with A-AAS (55.6 ± 16.7 versus 62.7 ± 13.1, *p =* 0.029). No significant differences (*p* > 0.05) in predisposing conditions were observed between the two groups in terms of whether or not combined with a history of high-risk underlying disease or presenting with high-risk clinical symptoms (violent and lasting chest, back or abdominal pain). However, in terms of physical findings, patients with A-AAS were more likely to have pulse deficit (36.7% versus 7.7%, χ^2^ = 22.74, *p =* 0.011), focal neurological deficit (with pain) (16.3% versus 3.8%, χ^2^= 8.23, *p* = 0.011) and hypotension or shock state (18.4% versus 7.7%, χ^2^ = 4.274, *p =* 0.039). A-AAS patients had more ADD markers (3.2 ± 1.5) than non-A-AAS patients (2.4 ± 1.6) when visiting the ED (*p* < 0.05).

### 3.2. Direct and Indirect Signs of the PHHE for the Diagnosis of A-AAS

**Figure 2:**
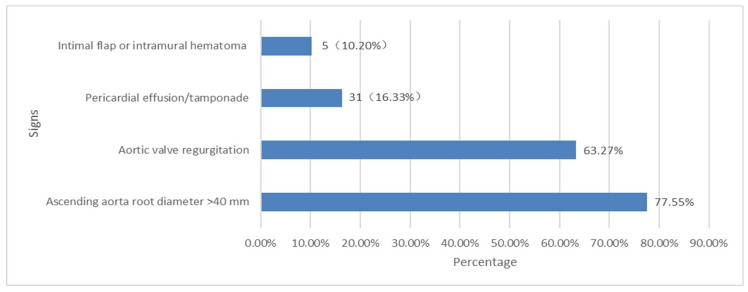
Proportion of PHHE signs in 49 A-AAS cases.

The direct signs (intimal flap or intramural hematoma of ascending aorta) and indirect signs (ascending aorta root diameter > 40 mm, pericardial effusion or tamponade and aortic valve regurgitation) detected by EM resident-performed FOCUS using PHHE are presented in Figure 2. Among all suspected patients, the most easily detectable sign by PHHE was dilation of the ascending aortic root. The positivity rate of ascending aortic root dilation in the A-AAS group was greater than that in the non-A-AAS group (incidence: 77.6% vs. 31.5%), and the differences were statistically significant (χ^2^ = 30.56, *p* = 0.013). The proportion of other positive detectable PHHE images occurred in the following order (from high to low; Figure 2): signs of aortic regurgitation, pericardial effusion/cardiac tamponade and intimal flap or intramural hematoma. Signs of pericardial effusion were detectable in 8 patients with A-AAS and 12 patients with non-A-AAS, but the difference was not significant (χ^2^ = 1.805, *p =* 0.179). The least easily detectable sign was the direct sign (intimal flap or intramural hematoma). In this study, a direct sign was reported in three patients with typical A-AD and two patients with IMH involving the ascending aorta and was falsely reported in three patients with another diagnosis in the non-A-AAS group. The difference between the two groups was statistically significant (χ^2^ = 5.197, *p* = 0.023). A “normal” PHHE was present in seven patients, with three patients ultimately diagnosed with A-AD, two patients diagnosed with A-IMH and two patients diagnosed with A-PAU. The clinical presentation and ADD scores of patients with A-AAS who were negative upon PHHE detection are shown in Table 2. To further analyze the characteristics of A-AAS patients with negative PHHE results, clinical feature findings were compared between the negative PHHE group and the positive group. Among all patients with A-AAS, the ADD score was significantly lower in the negative PHHE group (2.57 ± 0.98 and 3.71 ± 1.82, respectively; *p =* 0.028). The duration from onset to admission was also significantly shorter in the negative PHHE group (4.86 ± 1.70 h versus 7.23 ± 2.69, respectively; *p =* 0.028). Furthermore, more than half of the patients in the false-negative PHHE group (4/7, 57.1%) were ultimately diagnosed with A-PAU or A-IMH rather than typical A-AD.

### 3.3. Independent Diagnostic Performance of Each Sign of PHHE-FOCUS

The SE, SP, PPV, NPV and accuracy for each sign of PHHE-FOCUS are presented in Table 2. PHHE-detected direct signs of A-AAS (intimal flap/intramural hematoma) alone showed SE = 10.2%, SP *=* 97.7, PPV = 62.5% and NPV = 74.3%. According to indirect PHHE-FOCUS findings, dilation of ascending aortic alone showed SE = 77.6%, SP = 68.5%, PPV = 48.1% and NPV = 89%; aortic regurgitation had SE = 63.3%, SP =67.7%, PPV = 42.5% and NPV = 83%; and pericardial effusion/cardiac tamponade was associated with SE = 16.3%, SP = 90.8%, PPV = 40% and NPV = 74.2%.

**Figure 3:**
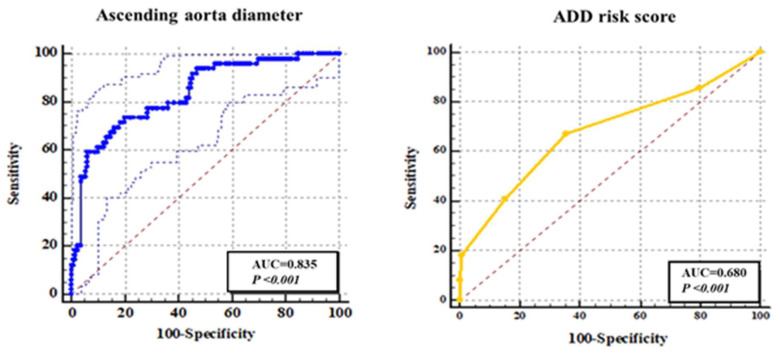
ROC curve showing the diagnostic performance of ADD score and ascending aorta diameter for A-AAS.

Because ADD score and the sign of ascending aortic dilatation were the most accessible indicators to EPs, we specifically evaluated their diagnostic efficacy, which is presented as the ROC curve separately in Figure 3. In the ROC curve showing the diagnostic performance of the aortic root diameter for A-AA, its AUC was 0.85. In addition, the AUC of ADD score was 0.68. When the ascending aorta diameter was 40.97 mm, which can be regarded as the optimal cutoff value for the diagnosis of A-AAS, the Youden index (YI) had a maximum value of 0.53. However, as shown in Figure 3, the optimal cutoff value of ADD score was 2 with a maximum YI value of 0.32 and maximum SE and SP values of 67.4% and 64.6%, respectively.

### 3.4. The Parallel and Sequence-Combined Diagnostic Value of PHHE-FOCUS Signs for A-AAS

A parallel combined diagnostic analysis of each imaging indicator of PHHE-FOCUS was performed using different diagnostic combined formulae. The overall SE, SP, PPV, NPV and AC of different parallel combination formulae are reported in Table 3. Accordingly, sequence combinations of PHHE-FOCUS diagnostic images are shown in Table 4. When conducting parallel combination tests, the SE of formulae 1 to 3 increased from 79.6% to 85.7%, whereas the SP and AC decreased instead. The sequence combination test showed a poor SE (4.1%) for the diagnosis of A-AAS, whereas no significant increase was observed in SP from formula 1 to formula 3 (Table 4).

A total of 19 hemodynamically unstable patients presenting with hypotension/shock at first visit were analyzed in this study, including 9 patients with A-AAS and 10 patients with non-A-AAS; the difference in critical patient population between the two groups was statistically significant (χ^2^ = 4.274, *p* = 0.039). The SE, SP, PPV and NPV of a positive PHHE direct sign were 55.6%, 100%, 100% and 71.4% in these 19 hypotension/shock patients, whereas the SE, SP, PPV and NPV of any sign of PHHE were 100%, 60%, 69.2% and 100%, respectively.

### 3.5. Diagnostic Efficiency of Acidic Calponin Alone or Combined with an Ascending Aorta Diameter >40 mm

**Figure 4:**
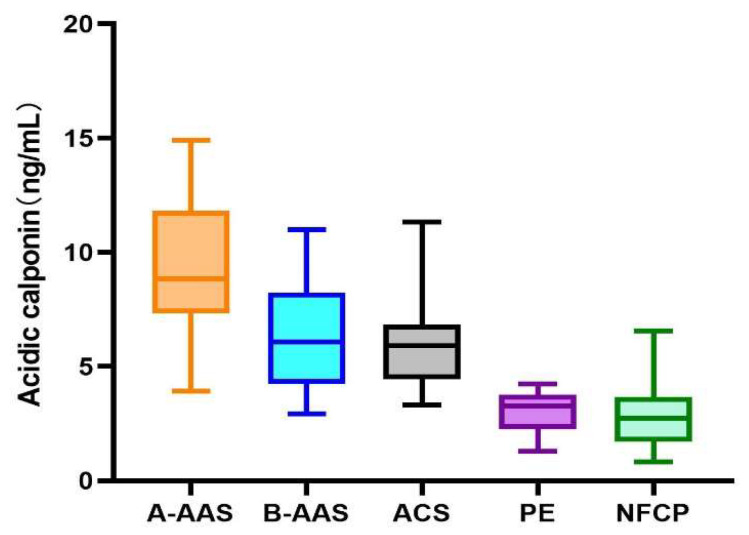
Acidic calponin levels in patients examined in the present study according to final diagnosis.

**Figure 5:**
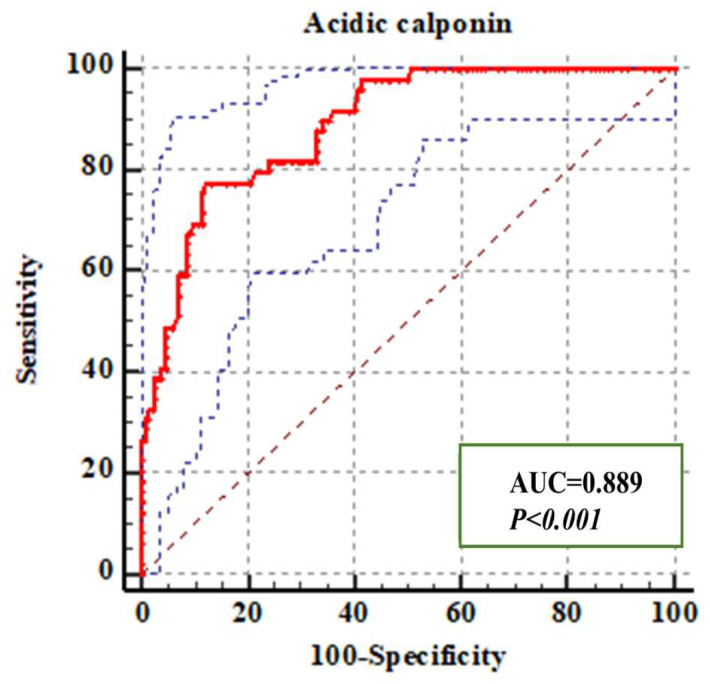
ROC curve showing the diagnostic value of acidic calponin for A-AAS.

We compared the acidic calponin levels (ng/mL) of A-AAS and non-A-AAS. The A-AAS group showed a significantly higher acidic calponin level than the non-A-AAS group (8.2 ± 1.21 versus 3.77 ± 1.12, respectively; *t* = 8.55, *p* < 0.05) at the time of admission. Furthermore, of the 179 suspected A-AAS cases, 49 (27.4%), 27 (15.1%), 36 (20.1%), 5 (2.8%) and 62 (34.6%) were categorized into A-AAS, B-AAS, ACS, PE and nonfatal chest pain (NFCP) groups, respectively, based on imaging and comprehensive clinical diagnosis. The NFCP group included pneumonia (*n* = 8), arteritis (*n* = 2), pericardial effusion (*n* = 12), neurological disease (*n* = 6), musculoskeletal disease (*n* = 8), digestive disease (*n* = 21) and other diagnoses (*n* = 5). Acidic calponin levels between different disease groups were significantly different (*p* < 0.05) (see Figure 4). Further analysis according to final diagnosis was performed for acidic calponin. As shown in Figure 4, AAS clearly showed elevations, with a marked increase in type A patients for acidic calponin. Acidic calponin levels were significantly higher in the A-AAS group than in the ACS (9.37 ± 2.85 versus 4.87 ± 1.48, *p =* 0.003), PE (9.37 ± 2.85 versus 3.06 ± 1.08, *p =* 0.032) and NFCP (9.37 ± 2.85 versus 2.06 ± 1.01, *p =* 0.001) groups, but there was no statistical difference between the A-AAS and B-AAS groups (9.37 ± 2.85 versus 6.35 ± 2.37, *p =* 0.265). The area under the ROC curve of acidic calponin for the diagnosis of A-AAS was 88.9%, with a cutoff value of 6.96 ng/mL, at which point the YI reached a maximum value of 0.6524, with SE and SP values of 77.6% and 87.7%, respectively (Figure 5).

**Figure 6:**
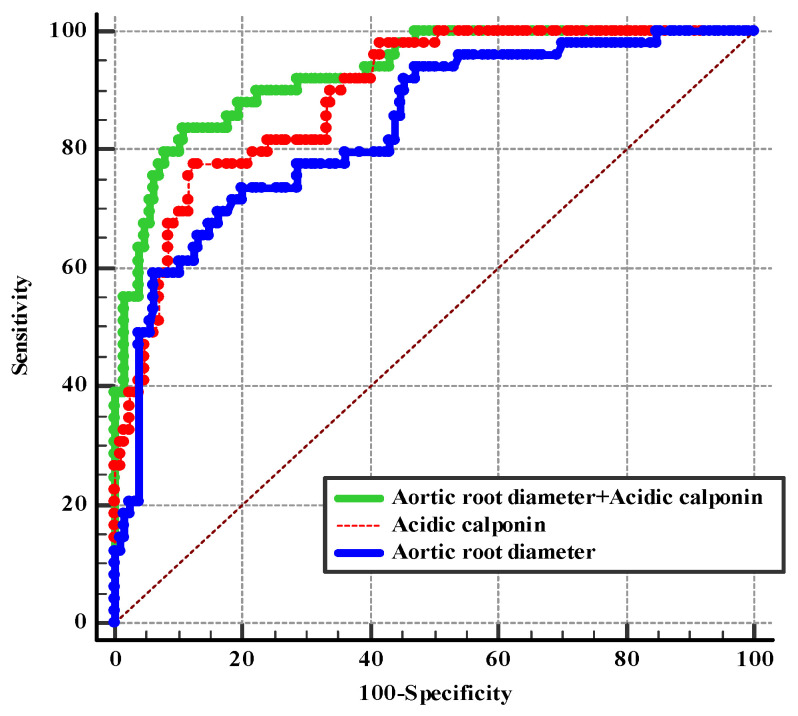
ROC curve showing the combined diagnostic performance of ascending aorta root diameter and acidic calponin for A-AAS.

Based on the diagnostic performance of PHHE previously described in this study, it was found that ascending aortic root dilation was the most easily detectable sign, with AUC > 70%. Meanwhile, the ascending aortic root diameter was the only quantifiable indicator of PHHE. Therefore, we next evaluated the performance of a diagnostic strategy combining ascending aortic root diameter with acidic calponin for A-AAS (Figure 6). The AUC of the combined indicators was 0.927, with a sensitivity and specificity of 83.7% and 89.2% (YI: 0.73), respectively. The combination of acidic calponin with an ascending aorta diameter >40 mm significantly improved diagnostic efficiency of A-AAS compared with either of them alone (*p =* 0.017, standard error 0.016, Z value 2.39; *p =* 0.001, standard error 0.028, Z value 3.29). However, there was no difference in the AUC of acidic calponin for the detection of A-AAS compared with the AUC of ascending aortic root diameter (*p* = 0.17, standard error = 0.04, Z-score = 1.38).

## 4. Discussion

A-AAS AAS is the most frequently fatal condition [27]. A common ED diagnostic dilemma is to differentiate between acute chest pain and other atypical symptoms and, consequently, avoiding inappropriate antithrombotic therapy and starting surgical intervention. Despite the development of imaging modalities including CTA, which is considered the current gold standard, CTA is still subject to many limits for several reasons [5,6]. Other bedside clinical tools for the early identification of A-AAS such as D-dimer and the ADD risk score have since gained attention. However, none of these tests considered singularly seem sufficiently accurate to promptly identify A-AAS, as each one shows an imbalance between sensitivity and specificity characteristics [28,29,30]. Thus, a convenient, quick and easy tool used by EM residents for the early detection of A-AAS is still needed. During the past two decades, the development of miniaturized ultrasound digital technology has advanced ultrasonic probes from echo rooms into the pockets of EPs’ white coats. Several studies have demonstrated the feasibility of PHHE, which offers advantages in terms of portability and speed when performed by clinicians. However, PHHE is considered to be inadequate for the diagnosis of A-AAS because of its suboptimal imaging quality, the subjective error of the naked eye and the skill level of inexperienced EPs [31,32]. Given these constraints, it is necessary to find suitable biomarkers of A-AAS that can help to improve the efficiency of A-AAS diagnosis and optimize the diagnostic strategies in the ED. We noticed that some studies [24,25,33] have suggested that calponin is of the same significance to smooth muscle as troponin is to the myocardium, the concentration of which increases in peripheral blood in AAS cases; therefore, acidic calponin could be of great value in the diagnosis of AAS. To the best of our knowledge, this is the first study evaluating the performance of a novel diagnostic strategy combining ADD risk score and PHHE with acidic calponin in a diagnostic approach to suspected A-AAS.

The detection of an intimal flap has been widely accepted as a helpful indicator in the diagnosis of A-AAS. Unfortunately, the SE of this direct PHHE sign was found to be substantially lower (10.2%) in our study than in a study by Cecconi and colleagues (87%) [29], who evaluated the diagnostic performance of FOCUS performed by well-trained cardiologists with long-standing experience, implying that this direct sign is difficult to discover in clinical practice, especially by EM residents who did not specialize in echocardiography. Intimal flap was incorrectly identified (false positive) in three patients ultimately diagnosed with non-A-AAS. The possible cause is that the operator misidentified calcified spots or artifacts as a torn intimal flap. Therefore, it is not acceptable to detect a cardiovascular emergency without delay by relying on this direct sign alone. In the present study, the diagnosis of A-AAS by EM resident-operated PHHE relied mostly on indirect signs (ascending aorta diameter >40 mm, pericardial effusion or tamponade and aortic valve regurgitation detected by color Doppler). Consist with the result of a previous study [30], an ascending aorta diameter >40 mm was found in 77.6% of A-AAS cases in our study, which was the most frequently detectable sign. This indirect sign was most easily obtained from the left parasternal view, which was best for visualizing the ascending aorta and the aortic root. EP residents could detect this sign immediately without postural restrictions and measurement of Doppler parameters. Thus, Wang et al. [34] recommended that PHHE should also be routinely available in ambulances and that EPs need to detect the ascending aorta diameter to screen A-AAD early and rapidly in patients presenting with acute chest pain. Furthermore, a ‘‘normal’’ PHHE (no sonographic signs of A-AAS) was present in seven patients with A-AAS, with more than 50% patients ultimately diagnosed with A-PAU or A-IMH instead of typical A-AD. The possible reasons are for this phenomenon include restricted body position, operator experience and skill and difficulty in detecting ulcer and hematoma caused by PAU/IMH. However, as previously shown, none of the indirect signs can be used as a stand-alone indicator to rule in or to rule out A-AAS in all suspected patients. Further parallel and sequence-combined diagnostic analyses of each imaging indicator of PHHE were conducted to find the best diagnostic combination. Although specificity reached up to 100%, the sequence-combined diagnostic sensitivity was only 4.1%, and the AC (73.2% to 73.7%) did not improve significantly. More importantly, a substantial number of A-AAS cases would have been missed if the decision had been based on the sequence-combined diagnosis test. Nonetheless, in our study, the presence of at least one PHHE sign (parallel combined diagnostic analysis) led to a substantially increased SE (4.1% to 85.7%), whereas the SP was attenuated from 100% to 61.5%. It is generally accepted that early detection of A-AAS and prompt initiation of emergent surgical intervention are crucial for the prognosis of critically ill patients who present with shock/hypotension upon first presentation. Therefore, we focused specifically on the diagnostic efficacy of PHHE in this cohort of patients. These findings indicate that the SP of direct signs of PHHE reached 100% in 19 critically ill patients with hypotension/shock, whereas detection of any PHHE signs showed 100% SE and 60% SP. These results are comparable to those reported in a study by Nazerian et al. [30], confirming that this sign represents a highly specific indicator suitable for the development of efficient rule-in diagnostic algorithms for A-AAS patients with unstable hemodynamics. We then investigated the possible reasons; in these patients, serious complications (dissection ruptured into pericardium, causing acute cardiac tamponade or severe aortic valvular insufficiency) developed, suggesting strong specificity. Accordingly, patients with shock/hypotension presenting with at least one PHHE sign of A-AAS are at great risk of long-distant transfer to CTA.

Because few studies have been conducted investigating acidic calponin testing per se for the diagnosis of A-AAS, special consideration has to be made for serological detection of suspected A-AAS patients; acidic calponin showed 77.6% SE and 87.7% SP with a cutoff value of 6.96 ng/mL. Its levels are elevated in the setting of both proximal and distal AAS within the first 24 h. As the only quantifiable and the most accessible PHHE indicator, an ascending aorta diameter equal to 40.97 mm can be regarded as the optimal cutoff value for the diagnosis of A-AAS, with an SE of 77.6% and an SP of 68.5% according to ROC curve analysis. In the present study, although acidic calponin increased specificity (68.5% to 87.7%) for the diagnosis of A-AAS compared with an ascending aorta diameter > 40 mm, the SE remained unchanged (77.6%). We therefore combined acidic calponin with an ascending aorta diameter > 40 mm to further investigate whether this combination could improve the diagnostic efficiency in the early identification of A-AAS. ROC curve analysis calculating the AUCs of indicators reported above confirmed the strongest diagnostic power of acidic calponin combined with an ascending aorta diameter > 40 mm, with an optimal SE of 83.7% and an SP of 89.2%. The combination of acidic calponin with an ascending aorta diameter > 40 mm significantly improved the diagnostic efficiency of A-AAS compared with either of them alone (*p =* 0.017, standard error 0.016, Z value 2.39; *p =* 0.001, standard error 0.028, Z value 3.29). In conclusion, it is possible to apply this indirect sign and acidic calponin as a novel candidate model for the early identification of A-AAS. However, calponin assays are not currently available as point-of-care tests in most emergency departments, as further technical improvements of the assay are necessary for production and commercial use.

Interestingly, in our study, we noted three distinctive highlights. One of the strengths of this study is that the diagnostic performance of the combined indicators was analyzed in suspected patients who were enrolled on the basis of ADD risk score and not by comparison with healthy controls. Thus, the diagnostic efficiency of combined detection reflects ‘real-world’ conditions. Secondly, previous research indicates a moderately high sensitivity and specificity when experienced investigators perform FOCUS [29,30]. Wang et al. [34] also conducted a study to evaluate the diagnostic performance of an ascending aorta diameter >40 mm for the early identification of A-AD. They suggested that further studies with the accuracy of PHHE routinely available in the ED or ambulance should also be conducted. Thus, in our study, we reported the diagnostic value of PHHE performed by frontline EM residents other than senior EPs or cardiologists in detecting A-AAS in the ED. The results show that for the early identification of A-AAS, indirect signs of PHHE are more applicable to general EPs and serve as a useful primary screening tool when combined with acidic calponin. IT is worth noting that PHHE can effectively rule out A-AAS in suspected patients with hypotension/shock when no signs are detected. PHHE can identify the ascending aorta diameter immediately and accurately, even when conducted by novice EPs. This result is also in line with a study of 239 A-AD cases in China recently reported by Wang et al. [34]. The final noteworthy strength of our study is that acidic calponin showed greater than twofold and threefold elevations compared with the other three groups of common emergencies (ACS, PE and NFCP) in the early period after symptom onset. For B-AAS, acidic calponin also showed an elevation that was not significantly different from that of the A-AAS group. These findings suggest that early accurate screening of A-AAS still requires combined diagnostic tools.

## 5. Limitations

This study is subject to several limitations, notably that it was a single-center retrospective observation. The population sample was relatively small. It is unclear whether the results can be generalized to other suspected patients with A-AAS. Secondly, the number of patients with shock/hypotension in this study was small, and further validation studies with a large sample are necessary. Thirdly, the diagnosis of A-AAS by transthoracic echocardiography remains a great challenge, even among experienced cardiologists, so the use of PHHE by EP is still a method with considerable limitations in clinical practice. Inexperienced users may be less able to provide optimal image quality than experts performing standard echocardiographic examination with PHHE, but we have no data to compare the test validity among such professionals.

## 6. Conclusions

Herein, we reported an initial study on the diagnostic value of EM resident-performed PHHE alone and combined with acidic calponin in patients with suspected A-AAS. The results of this preliminary study show that PHHE was highly indicative of A-AAS in patients presenting with shock or hypotension; however, further larger studies are warranted. An ascending aorta diameter >40 mm combined with acidic calponin demonstrated acceptable accuracy as a rapid first-line triage tool to identify patients with suspected A-AAS.

## Figures and Tables

**Table 1:** Baseline clinical characteristics and ADD markers of study patients.

	A-AAS (*n* = 49)	Non-A-AAS (*n* = 130)	*p*
**Male, *n* (%)**	35 (71.4%)	89 (68.5%)	0.701
**Median age (years)**	55.6 ± 16.7	62.7 ± 13.1	0.029
**High-risk underlying disease or conditions**			
Marfan syndrome (other connective tissue disease)	2 (4.1%)	0 (0%)	0.129
Family history of aortic disease	4 (8.2%)	10 (7.7%)	0.917
Known aortic valve disease	3 (6.1%)	8 (6.2%)	0.994
Known thoracic aortic aneurysm	6 (12.2%)	5 (3.8%)	0.082
Recent aortic manipulation	1 (2.0%)	0 (0%)	0.102
**High-risk pain features**			
Abrupt onset of pain	42 (85.7%)	118 (90.8%)	0.328
Severe pain intensity	37 (75.5%)	95 (73.1%)	0.741
Ripping/tearing pain	20 (40.8%)	46 (35.4%)	0.502
**High-risk vitals**			
Pulse deficit/systolic bloodpressure differential > 20 mmHg	18 (36.7%)	10 (7.7%)	0.011
Focal neurological deficit (accompanied by pain)	8 (16.3%)	5 (3.8%)	0.04
Murmur of aortic insufficiency (new onset)	8 (16.3%)	16 (12.3%)	0.482
Hypotension or shock state	9 (18.4%)	10 (7.7%)	0.039
**ADD markers**	3.2 ± 1.5	2.4 ± 1.6	0.001

**Table 2:** Diagnostic performance of each sign of PHHE-FOCUS.

	Sens, %	Spec, %	PPV, %	NPV, %	AC, %
**Direct sonographic signs**	10.2 (5/49)	97.7 (127/130)	62.5 (5/8)	74.3 (127/171)	73.7 (132/179)
**Ascending aortic dilatation**	77.6 (38/49)	68.5 (89/130)	48.1 (38/79)	89 (89/100)	70.9 (127/179)
**Pericardial effusion/tamponade**	16.3 (8/49)	90.8 (118/130)	40 (8/20)	74. (118/159)	70.4 (126/179)
**Aortic valve insufficiency**	63.3 (31/49)	67.7 (88/130)	42.5 (31/73)	83 (88/106)	66.5 (119/179)

**Table 3:** Parallel combined diagnostic performance of PHHE-FOCUS signs for A-AAS.

	Sens, %	Spec, %	PPV, %	NPV, %	AC, %
**Formula 1 ^a^**	79.6 (39/49)	67.7 (88/130)	48.1 (39/81)	89.8 (88/98)	70.9 (127/179)
**Formula 2 ^b^**	81.6 (40/49)	63.1 (82/130)	45.5 (40/88)	90.1 (82/91)	68.2 (122/179)
**Formula 3 ^c^**	85.7(42/49)	61.5 (80/130)	45.6 (42/92)	92 (80/87)	68.2 (122/179)

^a^ Any sonographic sign of direct sonographic signs + ascending aorta diameter > 40 mm. ^b^ Any sonographic sign of direct sonographic signs + ascending aorta diameter > 40 mm + pericardial effusion. ^c^ Any sonographic sign of direct sonographic signs + ascending aorta diameter > 40 mm + pericardial effusion + aortic valve regurgitation.

**Table 4:** Sequence-combined diagnostic performance of PHHE-FOCUS signs for A-AAS.

	Sens, %	Spec, %	PPV, %	NPV, %	AC, %
**Formula 1 ^d^**	4.1 (2/49)	99.2 (129/130)	66.7 (2/3)	73.3 (129/176)	73.2 (131/179)
**Formula 2 ^e^**	4.1 (2/49)	100 (130/130)	100 (2/2)	73.4 (130/177)	73.7 (132/179)
**Formula 3 ^f^**	4.1 (2/49)	100 (130/130)	100 (2/2)	73.4 (130/177)	73.7 (132/179)

^d^ Simultaneously positive: direct sonographic signs + ascending aorta diameter > 40 mm. ^e^ Simultaneously positive: direct sonographic signs + ascending aorta diameter > 40 mm + pericardial effusion. ^f^ Simultaneously positive: direct sonographic signs + ascending aorta diameter > 40 mm + pericardial effusion + aortic valve regurgitation.

## Data Availability

The data presented in this study are available on reasonable request from the corresponding author. The data are not publicly available for the privacy of individuals that participated in this study.
